# Plane-Stress Measurement in Anisotropic Pipe Walls Using an Improved Tri-Directional LCR Ultrasonic Method

**DOI:** 10.3390/s25144371

**Published:** 2025-07-12

**Authors:** Yukun Li, Longsheng Wang, Fan Fei, Dongying Wang, Zhangna Xue, Xin Liu, Xinyu Sun

**Affiliations:** 1 College of Pipeline and Civil Engineering Institute, China University of Petroleum (East China), Qingdao 266580, China; mliyk@upc.edu.cn (Y.L.); 20190007@upc.edu.cn (Z.X.); s23060015@s.upc.edu.cn (X.L.); z24060070@s.upc.edu.cn (X.S.); 2 PipeChina Beijing Gas Pipeline Co., Ltd., Beijing 100101, China; feifan@pipechina.com.cn (F.F.); wangdy13@pipechina.com.cn (D.W.)

**Keywords:** plane-stress state, longitudinal critical refraction wave, spiral-welded pipe, anisotropic, time-of-flight

## Abstract

It is important to accurately characterize the plane-stress state of pipe walls for evaluating the bearing capacity of the pipe and ensuring the structural safety. This paper describes a novel ultrasonic technique for evaluating anisotropic pipe-wall plane stresses using three-directional longitudinal critical refracted (LCR) wave time-of-flight (TOF) measurements. The connection between plane stress and ultrasonic TOF is confirmed by examining how the anisotropy of rolled steel plates affects the speed of ultrasonic wave propagation, which is a finding not previously documented in spiral-welded pipes. Then based on this relationship, an ultrasonic stress coefficient calibration experiment for spiral-welded pipes is designed. The results show that the principal stress obtained by the ultrasonic method is closer to the engineering stress than that obtained from the coercivity method. And, as a nondestructive testing technique, the ultrasonic method is more suitable for in-service pipelines. It also elucidates the effects of probe pressure and steel plate surface roughness on the ultrasonic TOF, obtains a threshold for probe pressure, and reveals a linear relationship between roughness and TOF. This study provides a feasible technique for nondestructive measurement of plane stress in anisotropic spiral-welded pipelines, which has potential application prospects in the health monitoring of in-service pipelines.

## 1. Introduction

The oil and gas industry extensively employs hot-rolled steel plates that comply with the American Petroleum Institute specifications to fabricate straight and spiral-welded pipelines [1]. These steel plates exhibit superior tensile strength and yield strength along the longitudinal direction (parallel to rolling orientation), while maintaining excellent ductility in the transverse direction (perpendicular to rolling axis) [2,3]. In spiral-welded pipe manufacturing, the coiling direction is typically oriented at 45° to 60° relative to the rolling direction. This process enables comprehensive utilization of the longitudinal and transverse mechanical properties of the steel plate, thereby mitigating stress concentration and alleviating anisotropic effects [4]. However, non-uniform material yielding during the rolling process introduces spatial variations in residual stress magnitude and orientation within the pipeline components [5,6,7]. Excessive stress concentrations can exacerbate girth-weld cracking susceptibility and compromise pipeline load-bearing capacity. Consequently, developing advanced plane-stress measurement methodologies for pipeline walls holds significant practical importance, providing critical technical support for the safety monitoring and structural integrity assessment of in-service pipelines. Some destructive methods can detect stress, but they are not suitable for in-service structures. Non-destructive testing (NDT) technology is a mature discipline developed in the late 20th century that is supported by international standard certification and professional academic research [8]. The NDE technique preserves the integrity of the initial structure while ensuring its precision and user-friendliness [9]. The nondestructive testing techniques for stress in in-service oil and gas pipelines mainly include X-ray diffraction [10,11,12], neutron diffraction [13,14,15], and magnetoelastic techniques incorporating Barkhausen noise and coercivity measurements [16,17,18,19], along with ultrasonic methods [20,21,22,23]. However, although ultrasonic testing is highly sensitive to surface and internal stress quantification through the principle of acoustoelasticity, it presents certain challenges in interpreting orientation-dependent stress–strain relationships, so its application in anisotropic materials is still in the exploratory stage.

Ultrasonic stress nondestructive testing employs diverse wave types, including surface waves [24,25], guided waves [26,27], shear waves [28], and longitudinal waves. Detection methods include the time-of-flight (TOF) [29,30] method and the nonlinear ultrasonic method [31,32]; the TOF method has been industrialized due to its simplicity and high accuracy and repeatability. Liu established a planar-stress Rayleigh-wave measurement model and calculated the planar stress of aluminum alloy plates after friction welding by measuring the time of Rayleigh-wave propagation in three directions [33]. Utilizing the birefringence concept of ultrasound transverse waves, Wang introduced longitudinal ultrasound waves along the thickness of a pipe, expanded the stress velocity equations, and successfully measured plane stress [34,35]. Among ultrasonic modes, longitudinal waves exhibit superior stress sensitivity and propagation velocity, particularly the ultrasonic longitudinal critical refraction wave (LCR wave). Generated at the first critical angle, LCR waves combine directional precision with stress responsiveness, enabling surface stress evaluation in operational structures. Pioneered by Bray for residual stress detection in heat-treated welded steel [36,37], the LCR method has shown a strong correlation with strain gauge measurements. Subsequent applications include Javadi measuring austenitic stainless steel tube-weld stresses [38] and Sadeghi assessing residual stress distribution in aluminum welds using frequency-variable LCR waves [39]. The above results show that the existing research mainly address the uniaxial stress state through velocity–stress correlations. Actual pipeline scenarios involve a complex plane-stress field, in which the principal stress orientations are indeterminate; thus, it would be interesting to know how to measure the principal stress of anisotropic materials.

Currently, assessing plane stresses in anisotropic substances through the ultrasonic LCR wave technique is predominantly utilized in composite materials. The relationship between TOF and stress–strain in composites was experimentally investigated by AA dos Santos et al. [40]. The propagation velocity, temperature effect, and acoustic elasticity coefficient were evaluated for different fiber orientations, with the most significant change in fiber orientation due to the speed of sound. At present, a mature sound velocity–stress constitutive model has been established using ultrasonic LCR wave detection technology for plane stress in composite materials [41,42], and the quantitative characterization of the multiaxial stress state of composite material has been successfully realized based on the anisotropic propagation characteristics of LCR waves [43,44]. Steel plates have longer grain sizes along the rolling direction and smaller grain sizes perpendicular to the rolling direction, similar to the structural differences between fiber-oriented and non-fiber-oriented directions in fiber-reinforced composites, leading to variations in mechanical properties and ultrasonic sound velocity across different directions of the steel plate and composite materials. Therefore, hot-rolled steel plates share similar anisotropic characteristics with fiber-reinforced composites [45,46,47], but measuring the stress in the walls of steel-plate pipes still faces challenges: (1) The curvature effect of the pipe wall causes the distortion of the LCR wave propagation path, and the repetitive coupling of the traditional unidirectional probes will inevitably lead to the accumulation of measurement errors and reduce the reliability of the stress measurement. (2) The spatial heterogeneity of stress coefficients triggered by the anisotropy of steel plate rolling increases the complexity of calibration experiments. (3) The influence of probe extrusion pressure and surface roughness on the anisotropic stress measurement method is not clear. To our knowledge, there is no relevant research on the above-mentioned issues in the existing literature. And the above issues will be analyzed in depth in this paper.

Based on the acoustoelasticity theory, a tri-directional ultrasonic LCR wave probe is designed to measure the TOF under the same coupling condition. By considering the anisotropy of a rolled steel plate, the calibration experiment of the stress coefficient of a spiral-welded pipe is conducted. The stress values measured by the ultrasonic method and the coercivity method are compared with the engineering stress values. And this paper also compares the stress values measured by the ultrasonic method with that obtained by the hole-drilling method for off-line pipelines. Finally, the influence of probe pressure and surface roughness of a steel plate on the TOF is studied. The results show that the improved ultrasonic method is more suitable for the stress measurement of in-service pipelines.

## 2. Principle of the Plane-Stress Measurement

### 2.1. The Relationship Between Plane Stress and the Velocity of Sound

**Figure 1:**
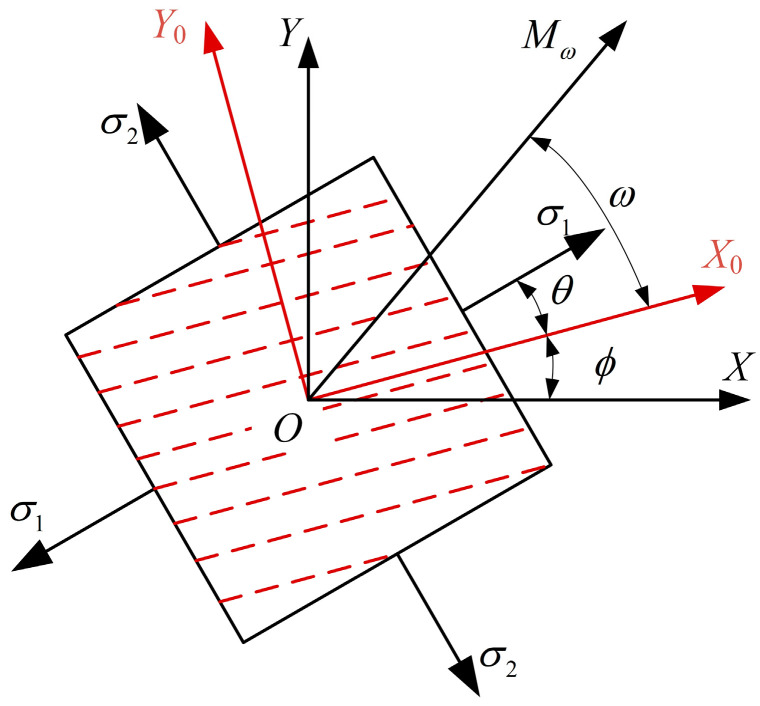
Coordinate system for the test of ultrasonic stress in anisotropic materials.

Regarding the rolled steel plate, the coordinate system depicted in Figure 1 is designated for analysis, taking into account the angular discrepancy between the direction of rolling and the principal stress direction of the steel plate. The shaded region in the figure represents a micro-element of the pipe wall surface, while the coordinate axes and angular directions are explicitly indicated. (1) Define the global coordinate system: on the unidirectional tensile specimen, the tensile direction is the *x*-axis, and the perpendicular to the tensile direction is the *y*-axis; on the wall of the pipe, the pipeline’s axial direction is the *x*-axis, and the tangential direction is the *y*-axis. (2) The main coordinate system is *X*_0_*OY*_0_, in which the *X*_0_ direction is the rolling direction of steel plate and *Y*_0_ is perpendicular to the rolling direction of steel plate. (3) The angle between the rolling direction and the first principal stress direction is *θ*, and the angle between the rolling direction and the *X* direction is ϕ. For a spiral-welded pipe, ϕ corresponds to the spiral angle. (4) The ultrasonic detection is performed along direction $M_{\omega }$, where *ω* denotes the angle between $M_{\omega }$ and the *X*_0_-axis.

When there is no residual stress inside the pipeline steel, that is, in the natural state, the main direction is specified to be the same as the rolling direction of the steel plate. When a micro-element is in a plane-stress state and undergoes deformation, the microscopic grain size of the material changes under such deformation conditions, and the principal direction also alters accordingly. Ultrasonic vibration is introduced in the deformation state, when the steel plate is in the detection state. In the process of changing from the natural state to the detection state, between the ultrasonic LCR wave propagation velocity change and the stress exists a linear relationship in the form of sound velocity matrix $\left[V_{\mathrm{ij}}\right]$, which can be expressed as follows:

(1) $$V_{ij}={\left(V_{ij}\right)}_{0}+\Delta V_{ij},\;\Delta V_{ij}=\alpha_{ij}\sigma_{kl}$$

where $\left[a_{\mathrm{ij}}\right]$ symbolizes the matrix of stress coefficients that establishes the orthogonal, which is analogous to the flexibility matrix defined in the following form:

(2) $$\left[\alpha_{ij}\right]=\left[\begin{array}{cc} \begin{array}{ccc} \alpha_{11} & a_{12} & \alpha_{13}\\ \alpha_{12} & \alpha_{22} & \alpha_{23}\\ \alpha_{13} & \alpha_{23} & \alpha_{33} \end{array} & 0\\ 0 & \begin{array}{ccc} \alpha_{44} & & \\ & \alpha_{55} & \\ & & \alpha_{66} \end{array} \end{array}\right]$$

For the plane-stress problem, $\sigma_{z}={\;\tau }_{\mathrm{xz}}={\;\tau }_{\mathrm{yz}}=0$, so the stress vector is as follows:

(3) $$\left\{\sigma_{kl}\right\}=\left\{\begin{array}{c} \sigma_{x}\\ \sigma_{y}\\ 0\\ \tau_{xy}\\ 0\\ 0 \end{array}\right\}$$

Mapping the results of $\alpha_{ij}\sigma_{kl}$ to a 2 × 2 mesoscopic tensor, the relationship between the sound velocity change matrix and the stress can be expressed as follows:

(4) $$\left[\Delta V_{ij}\right]=\left[\begin{array}{cc} \Delta V_{x} & \Delta V_{xy}\\ \Delta V_{xy} & \Delta V_{y} \end{array}\right]=\left[\begin{array}{cc} \alpha_{11}\sigma_{x}+\alpha_{12}\sigma_{y} & \alpha_{44}\tau_{xy}\\ \alpha_{44}\tau_{xy} & \alpha_{12}\sigma_{x}+\alpha_{22}\sigma_{y} \end{array}\right]$$

Substituting Equation (4) into Equation (1) yields the following:

(5) $$\left[V_{ij}\right]=\left[\begin{array}{cc} V_{X_{0}} & \\ & V_{Y_{0}} \end{array}\right]+\left[\begin{array}{cc} \Delta V_{x} & \Delta V_{xy}\\ \Delta V_{xy} & \Delta V_{y} \end{array}\right]=\left[\begin{array}{cc} V_{X_{0}}+\alpha_{11}\sigma_{x}+\alpha_{12}\sigma_{y} & \alpha_{44}\tau_{xy}\\ \alpha_{44}\tau_{xy} & V_{Y_{0}}+\alpha_{12}\sigma_{x}+\alpha_{22}\sigma_{y} \end{array}\right]$$

where $V_{X_{0}}$, $V_{X_{0}}$ is the ultrasonic propagation velocity along the rolling direction and perpendicular to the rolling direction in the natural state.

The principal values of the sound velocity matrix $\left[V_{\mathrm{ij}}\right]$ in the principal direction for the plane-stress state are as follows:

(6) $$V_{X,Y}=\frac{1}{2}\left\{\left[V_{X_{0}}+V_{Y_{0}}+\left(\alpha_{11}+\alpha_{12}\right)\sigma_{x}+\left(\alpha_{12}+\alpha_{22}\right)\sigma_{y}\right]\pm \sqrt{{\left[V_{X_{0}}-V_{Y_{0}}+\left(\alpha_{11}-\alpha_{12}\right)\sigma_{x}+\left(\alpha_{12}-\alpha_{22}\right)\sigma_{y}\right]}^{2}+4{\alpha_{44}}^{2}{\tau_{xy}}^{2}}\right\}$$

The angle between the rolling direction and the *X* direction is as follows:

(7) $$\tan2\phi =\frac{2\alpha_{44}\tau_{xy}}{\left(V_{X_{0}}-V_{Y_{0}}\right)+\left(\alpha_{11}-\alpha_{12}\right)\sigma_{x}+\left(\alpha_{12}-\alpha_{22}\right)\sigma_{y}}$$

According to the knowledge of mechanics of materials, the conversion relationship between the stress in any direction and the principal stress is as follows:

(8) $$\left\{\begin{array}{c} \sigma_{x}=\frac{1}{2}\left[\sigma_{1}+\sigma_{2}+\left(\sigma_{1}-\sigma_{2}\right)\cos2\theta \right]\\ \sigma_{y}=\frac{1}{2}\left[\sigma_{1}+\sigma_{2}-\left(\sigma_{1}-\sigma_{2}\right)\cos2\theta \right]\\ \tau_{xy}=\frac{1}{2}\left(\sigma_{1}-\sigma_{2}\right)\sin2\theta \end{array}\right.$$

The sound velocity matrix $\left[V_{\mathrm{ij}}\right]$ has similar vector decomposition properties as the stress matrix, so that the sound velocity in any direction in the plane-stress state is as follows:

(9) $$V_{\omega }=\frac{1}{2}\left(V_{X}+V_{Y}\right)+\frac{1}{2}\left(V_{X}-V_{Y}\right)\cos2\left(\omega -\phi \right)$$

Substituting Equations (6) and (7) into Equation (9) yields the following:

(10) $$\begin{array}{c} V_{\omega }=\frac{1}{2}\left[V_{X_{0}}+V_{Y_{0}}+\left(\alpha_{11}+\alpha_{12}\right)\sigma_{x}+\left(\alpha_{12}+\alpha_{22}\right)\sigma_{y}\right]+\alpha_{44}\tau_{xy}\sin2\omega \\ +\frac{1}{2}\left[V_{X_{0}}-V_{Y_{0}}+\left(\alpha_{11}-\alpha_{12}\right)\sigma_{x}+\left(\alpha_{12}-\alpha_{22}\right)\sigma_{y}\right]\cos2\omega \end{array}$$

When the material is not stressed, Equation (9) is rewritten as follows:

(11) $$V_{\omega ,0}=\frac{1}{2}\left(V_{X_{0}}+V_{Y_{0}}\right)+\frac{1}{2}\left(V_{X_{0}}-V_{Y_{0}}\right)\cos2\left(\omega -\phi \right)$$

Equations (9) and (11) represent the quantitative relationship between ultrasonic propagation velocity and stress before and after the material is subjected to plane stress, respectively.

Through the Equations (9) and (11), we can establish a correlation between the alteration in sound speed and the principal stresses along any axis in the plane-stress condition:

(12) $$\begin{array}{cc} \Delta V_{\omega } & =V_{\omega }-V_{\omega ,0}=\frac{1}{4}\left(\alpha_{11}+\alpha_{22}+2\alpha_{12}\right)\left(\sigma_{1}+\sigma_{2}\right)\\ & +\frac{1}{4}\left(\alpha_{11}-\alpha_{22}\right)\left(\sigma_{1}-\sigma_{2}\right)\cos2\theta +\frac{1}{4}\left(\alpha_{11}-\alpha_{22}\right)\left(\sigma_{1}+\sigma_{2}\right)\cos2\omega \\ & +\frac{1}{4}\left(\alpha_{11}+\alpha_{22}-2\alpha_{12}\right)\left(\sigma_{1}-\sigma_{2}\right)\cos2\theta \cos2\omega +\frac{1}{2}\alpha_{44}\left(\sigma_{1}-\sigma_{2}\right)\sin2\theta \sin2\omega \end{array}$$

### 2.2. Principal Stress Measurement Method of Pipe Walls

The method of measuring the plane stresses in the material given in Equation (12) is difficult in practice due to the fact that it is difficult to accurately measure the ultrasonic propagation speed of sound. By fixing the distance between the ultrasonic transmitting and receiving transducers, the ultrasonic TOF can be measured accurately, which is a relatively simple method with high measurement accuracy.

The time required for an ultrasonic wave to propagate a fixed distance can be calculated from the ratio of the distance to the speed of sound. Since the time used by the ultrasonic wave to propagate the distance covered by the probe is very small, full differentiation of the time yields a linear relationship between the two small changing quantities:

(13) $$\frac{\Delta t}{\Delta V}=-\frac{L}{V^{2}}$$

where ∆*t* represents the amount of change in ultrasonic TOF, Δ*V* represents the small variation in sound speed from its natural state (sound speed as $V_{0}$) to the plane-stress state (sound speed as *V*), and *L* represents the distance traveled by the ultrasonic waves.

Let $K_{0}=-\left(L/V^{2}\right)$, then Equation (9) can be written as follows:

(14) $$t=K_{1}\sigma_{1}+K_{2}\sigma_{2}$$

where

(15) $$\begin{array}{l} K_{1}=p_{1}\left(\cos2\omega +\cos2\theta \right)+p_{2}+p_{3}\cos2\left(\omega -\theta \right)+p_{4}\cos2\left(\omega +\theta \right)\\ K_{2}=p_{1}\left(\cos2\omega -\cos2\theta \right)+p_{2}-p_{3}\cos2\left(\omega -\theta \right)-p_{4}\cos2\left(\omega +\theta \right)\\ p_{1}=K_{0}\left(\alpha_{11}-\alpha_{22}\right)/4\\ p_{2}=K_{0}\left(\alpha_{11}+\alpha_{22}+2\alpha_{12}\right)/4\\ p_{3}=K_{0}\left(\alpha_{11}+\alpha_{22}-2\alpha_{12}+2\alpha_{44}\right)/8\\ p_{4}=K_{0}\left(\alpha_{11}+\alpha_{22}-2\alpha_{12}-2\alpha_{44}\right)/8 \end{array}$$

As per Equation (14), under plane stress, the ultrasonic TOF in a single direction is simultaneously influenced by the two principal stresses, with the *K*_1_ and *K*_2_ coefficients correlating to the principal stress direction *θ* and the ultrasonic propagation direction *ω*.

During the actual measurement phase, the direction of ultrasonic propagation ω is typically established, leading to three unidentified variables in Equation (14): *σ*_1_, *σ*_2_, and *θ*. Consequently, assessing the ultrasonic transmission time in a minimum of three orientations is crucial for finding an accurate solution to the equation system and determining the principal stresses’ intensity and orientation.

In order to simplify the measurement, three directions are chosen to measure the ultrasonic TOF: the axial direction of the pipe, the 45° direction, and the annular direction of the pipe. When the pipe is a spiral-welded pipe, the angle between the axial direction of the pipe and the rolling direction of the steel plate is the spiral angle ϕ. For the convenience of arranging tri-directional combination probes, the angle between two adjacent detection directions is specified to be 45°, such that $\omega_{1}=\phi$, $\omega_{2}=\phi +45{}^{\circ}$, $\omega_{3}=\phi +90{}^{\circ}$.

## 3. Experiment

**Figure 2:**
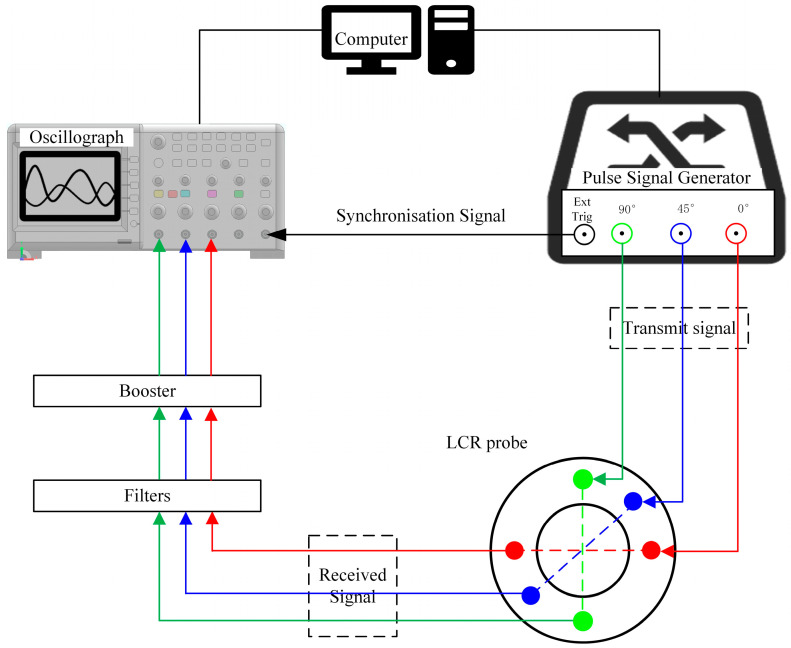
Measurement system of an ultrasonic critical refraction longitudinal wave.

For the ultrasonic LCR wave measurement system, as shown in Figure 2, the high-voltage pulse signals excited by the signal generator are transmitted to the ultrasonic transmitting transducer, while synchronous signals are generated and sent to the digital oscilloscope. The transmit transducer converts the received high-voltage pulse signal into mechanical vibration. The vibrations propagate in the form of longitudinal waves on the surface of the specimen to be tested. Upon receiving the vibration, the receiving transducer converts the vibration signal into an electrical signal, which is then digitized. After passing through a filter and preamplifier, the signal is transmitted to the digital oscilloscope. The digital oscilloscope displays the time domain diagram of the ultrasonic waveform, with the horizontal axis representing the time delay of the vibration signal (i.e., the ultrasonic propagation time) and the vertical axis indicating the amplitude of the vibration signal. The computer processes the LCR wave feature point coordinates to determine stress values. To achieve an ultrasonic propagation time measurement accuracy of 0.5 ns, the oscilloscope sampling rate is set to 2 GS/s.

**Figure 3:**
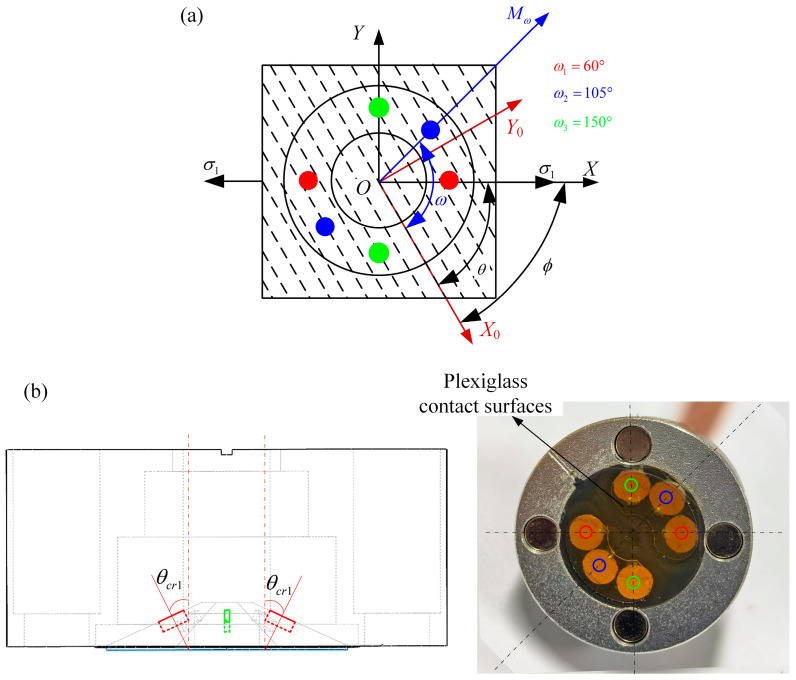
Tri-directional ultrasound probe: (**a**) detection orientation and (**b**) probe structure.

As shown in Figure 3a, three directional ultrasound TOF signals are measured at once in the same coupling state using a tri-directional probe. The angle between two adjacent directions is 45°, and one direction includes a transmitting transducer and a receiving transducer. The red, blue, and green probes correspond to the ultrasonic detection directions $\omega_{1},{\;\omega }_{2},{\;\omega }_{3}$, respectively. The center frequency of the piezoelectric wafer inside the transducer is 5 MHz, and the diameter of the wafer is 6 mm. As shown in Figure 3b, the ultrasonic incidence angles in three directions are equal to the first critical refraction angle ($\theta_{\mathrm{cr}1}=28.5{}^{\circ}$), which is calculated by measuring the sound velocity of the steel plate and the probe wedge and bringing it into Snell’s law formulation. The plexiglass contact surface at the bottom of the transducer is optimized, with the aim of reducing the coupling contact area and avoiding measurement errors caused by uneven grinding of the measured surface. Silicone oil with a kinetic viscosity of 1000 cst is used as a coupling agent between the transducer and the measured steel pipeline specimen. Two sets of magnets symmetrically distributed along the center of the probe are used to fix the probe to the surface of the steel pipe specimen to ensure that the direct squeezing force between the probe and the measured material is constant. It is important to ensure that the ambient temperature is 25 °C and the surface temperature of the specimen is constant during the measurement.

In order to avoid the interference of abnormal data and ensure that the correlation coefficient of the linear relationship of the coefficient calibration experiment is greater than 0.99, the ultrasonic TOF signal was measured five times repeatedly under the same loading conditions.

## 4. Results and Discussion

### 4.1. Results of Calibration

**Figure 4:**
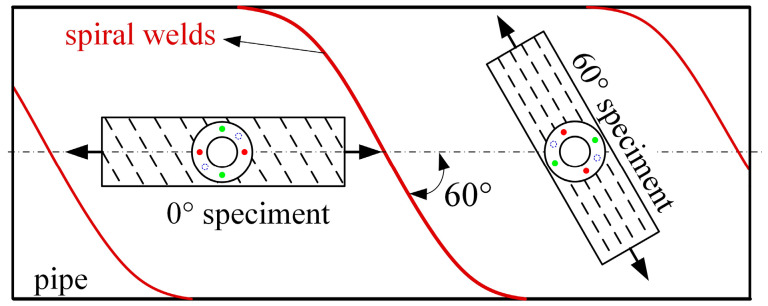
Schematic diagram of the calibration specimen preparation direction.

The calibration specimens are unidirectional tensile specimens, which are processed and prepared from X80 spiral-welded steel pipe with a helix angle of 60°, and two kinds of tensile specimens are prepared along the axial direction of the pipe and the rolling direction, respectively, as shown in Figure 4. As shown in Table 1, two specimens of each type are selected as calibration specimens for fitting the stress coefficients. An additional 0° orientation specimen is machined as a uniaxial tensile verification test specimen. All specimens are annealed with heat to eliminate internal residual stresses.

In the uniaxial tensile stress state, *σ*_1_ = *σ* and *σ*_2_ = 0. By measuring the ultrasonic TOF in different directions and substituting into Equations (14) and (15), four equations about *p*_i_ are obtained, which can be solved for *p*_1_, *p*_2_, *p*_3_, and *p*_4_ after association.

Four groups of uniaxial tensile tests are carried out using specimens numbered exp_1, exp_2, exp_4, and exp_5. The ultrasonic TOF is measured under different tensile load conditions, and the linear relationship between the ultrasonic TOF and the principal stress $\sigma_{1}$ is fitted. Among them, the specimens numbered exp_1 and exp_4 measured the ultrasonic TOF in the tensile direction during the stretching process, while the specimens numbered exp_2 and exp_5 measured the ultrasonic TOF perpendicular to the tensile direction during the stretching process. The calibration state and angle combinations are shown in Table 2. Apply uniaxial tensile load to the specimen, starting from 0 stress; the upper limit of stress loading is 450 MPa, and every 50 MPa for a hold load, repeat the coupling probe at each hold load stage and measure the ultrasonic TOF.

**Figure 5:**
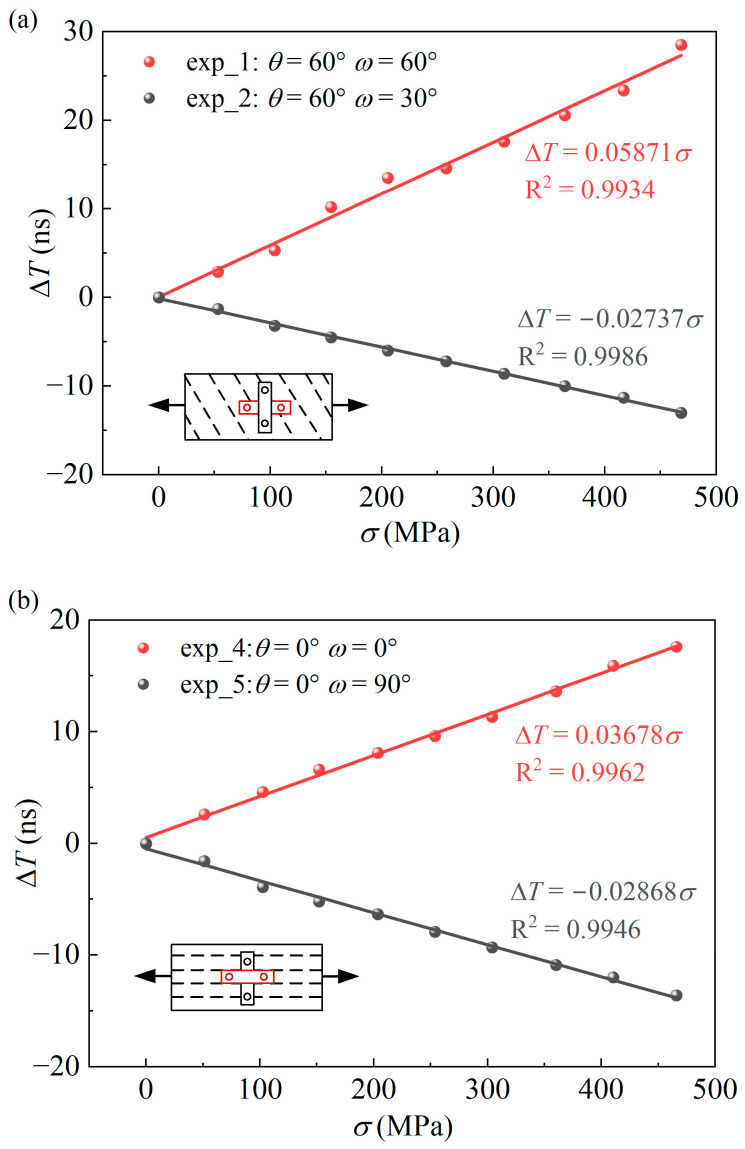
Fitting results of a stress coefficient calibration, measured along and perpendicular to the tensile direction: (**a**) Axial calibration specimens: exp_1 and exp_2. (**b**) Calibration specimens along the rolling direction: exp_4 and exp_5.

The ultrasonic TOF measurements of the four groups of calibration experiments under different stress states are shown in Figure 5. According to the change of ultrasonic TOF with stress in the figure, it can be seen that the ultrasonic TOF measured along the tensile direction increases linearly with the increase of tensile stress, and the ultrasonic TOF measured perpendicular to the tensile direction decreases linearly with the increase of tensile stress. On the one hand, the linear relationship is a manifestation of the acoustoelastic effect, that is, the increase in stress causes a change in the speed of sound, which confirms the existing theoretical model. On the other hand, the anisotropy of the steel plate leads to different linear fitting results. The micro-grain organization grows along the tensile direction, and the ultrasonic sound velocity slows down under the action of attenuation, resulting in an increase in the ultrasonic TOF, while perpendicular to the stretching direction is the exact opposite.

Table 3 displays the outcomes of slope fitting across four experimental groups, and these slopes are integrated into the stress coefficient formula in Table 2 for resolving the equation system, yielding the outcomes for *p*_1_ to *p*_4_ as follows: *p*_1_ = −0.04648, *p*_2_ = 0.05053, *p*_3_ = 0.00087, and *p*_4_ = −0.07833. From this, planar stress can be measured based on this ultrasonic stress factor calibration result.

On the surface of the material with a known rolling direction, the ultrasonic propagation time is measured using a three-directional ultrasonic probe. Based on Equation (14), a nonlinear system of equations is established regarding the principal stresses *σ*_1_ and *σ*_2_ and the principal stress direction *θ*. All other variables, including the ultrasonic TOF and ω, are known, except for the principal stresses. Based on the calibration results of coefficients *p*_1_ to *p*_4_, the nonlinear system of equations can be solved to determine the principal stresses.

### 4.2. Results of Verification

In this paper, the ultrasonic measurement method of plane stress is verified by uniaxial tensile stress measurement and pipe-wall stress measurement.

**Figure 6:**
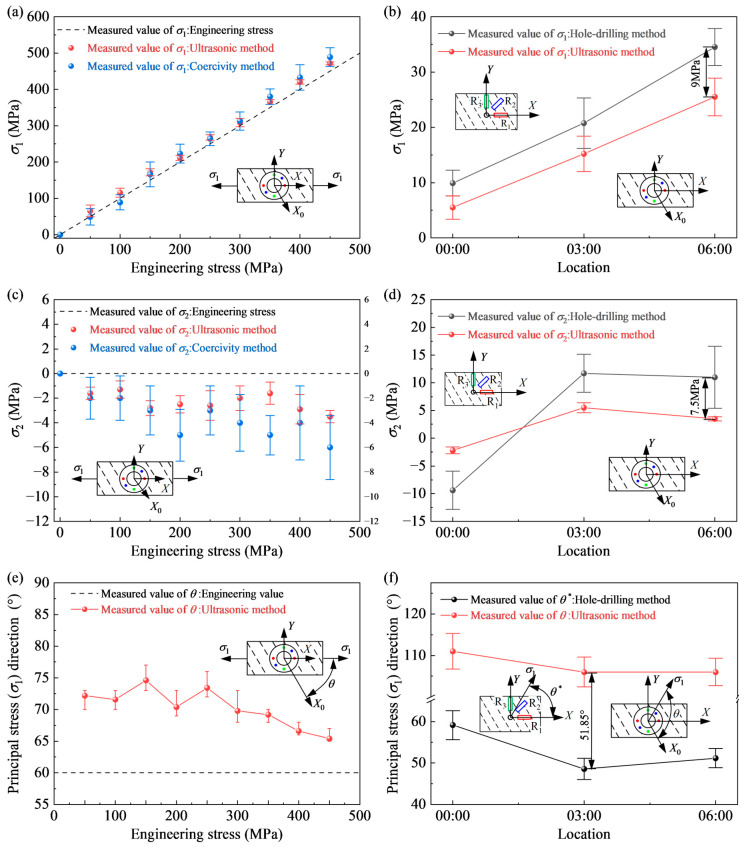
Measurement results of $\sigma_{1}$, $\sigma_{2}$, and θ: (**a**) Results of $\sigma_{1}$ from the uniaxial tensile experiment. (**b**) Results of $\sigma_{1}$ from the pipe experiment. (**c**) Results of $\sigma_{2}$ from the uniaxial tensile experiment. (**d**) Results of $\sigma_{2}$ from the pipe experiment. (**e**) Principal stress $\sigma_{1}$ direction from the uniaxial tensile experiment. (**f**) Principal stress $\sigma_{1}$ direction from the pipe experiment.

A uniaxial tensile load is applied to specimen exp_3, starting from 0 stress, with a stress loading limit of 450 MPa and holding loads at 50 MPa intervals. The ultrasonic TOF of the specimen is measured at each holding stage by using the tri-directional probes. Set the ultrasonic testing direction $\omega_{1}$ to follow the tensile direction. In this case $\omega_{1}=60{}^{\circ}$, $\omega_{2}=105{}^{\circ}$, and $\omega_{3\;}=150{}^{\circ}$. At the same load level, the maximum fluctuation of ultrasonic TOF measurement time was ±1.33 ns. The TOF measurement results with errors are brought into the MATLAB (R2023b) nonlinear equation solver, and the solutions (*σ*_1_, *σ*_2_, and *θ*) under different errors are calculated. In order to compare the accuracy of this method with other non-destructive testing techniques for stress, during the load holding phase, in addition to using the ultrasonic method, the coercive force method is also used to measure the principal stress $\sigma_{1}$ along the tensile direction. The stress test results of the exp_3 are shown in Figure 6.

It can be seen from Figure 6a,c that the first principal stress $\sigma_{1}$ measured by the ultrasonic method increases linearly with the increase of load, and the standard deviation is 20.5 MPa, which is smaller than that measured by the coercivity method and is closer to the engineering stress. The second principal stress value measured by the ultrasonic method is compressive stress, and the compressive stress slightly increases with the increase of tensile load, but it is still relatively small. This is due to the decrease in grain size perpendicular to the stretching direction during the stretching process, resulting in an increase in ultrasonic propagation speed, rather than an increase in compressive stress.

The results of the principal stress direction θ measured by the ultrasonic method in specimen exp_3 are shown in Figure 6e. The mean value of the principal stress direction fluctuates in the range of 65–77°, and the maximum standard deviation is 1.85°. The deviation from the theoretical value is 5–17° (8.3~28.3%). Given that the specimen exp_3’s rolling and tensile directions intersect at an angle of 60°, the theoretical outcome of θ is 60°. With the increase of tensile load, the principal stress direction is getting closer and closer to the tensile direction. The observed fluctuations arise because the ultrasonic measurement direction is not strictly aligned with the nominal 0°, 45°, and 90° directions. This misalignment introduces deviations in the ultrasonic TOF measurements, which propagate into the solution of the nonlinear equations, causing divergence and amplifying the error in the calculated angle θ. An additional plausible explanation could be the angular difference between the specimen’s tensile and long orientations.

In this paper, an X80 pipe section with a helix angle of 60° is taken as the experimental object, the 9:00 position is the intersection of the spiral weld and the girth weld, and the residual stresses of the pipe wall near the girth weld at 0:00, 3:00, and 6:00 are measured by the ultrasonic method and the hole-drilling method. The stress measurement point is located 25 mm from the center of the girth weld on one side of the weld to avoid interference from the heat-affected zone of the girth weld. Firstly, the plane stress of the pipe wall is measured by the ultrasonic method. Set the ultrasonic testing direction $\omega_{1}$ to follow the axial direction of the pipe, where $\omega_{1}=60{}^{\circ}$, $\omega_{2\;}=105{}^{\circ}$, $\omega_{3}=150{}^{\circ}$. The principal stress ($\sigma_{1}$) direction calculated by the ultrasonic method is denoted as θ, that is, the angle between $\sigma_{1}$ and the rolling direction. Then drill holes in the center of the ultrasonic stress measurement point and measure the strain change at the edge of the hole after drilling. Use a microscope to position the center position before drilling to improve the accuracy of alignment. The strain release coefficient was obtained through the calibration experiment with the X80 steel. The experimental results A = −0.56 με/MPa and B = −0.106 με/MPa were used in this study. In this experiment, the error of the blind hole stress measurement only takes into account the offset of the strain reading. The paste direction of the three strain gauges is the same as that of the three ultrasonic detection directions. The principal stress ($\sigma_{1}$) direction calculated by the hole-drilling method is denoted as $\theta^{*}$, that is, the angle between $\sigma_{1}$ and the axial direction of the pipeline. Therefore, theoretically, there is a difference of exactly 60° between $\theta^{*}$ and θ.

The stress measurement results of the ultrasonic method and the hole-drilling method in three stress measurement areas are shown in Figure 6b,d,f. The two principal stresses measured by the ultrasonic method have the same sign as those measured by the hole-drilling method, and their trends with the change of clock position are consistent. Compared with the stress measurement results of the hole-drilling method, the principal stress measured by the ultrasonic method is generally smaller (or the compressive stress is larger), with a maximum deviation of about 9 MPa. It can be attributed to the fact that although the center of the ultrasonic stress measurement point coincides with the drilling position, the ultrasonic method measures the average stress value within the area enclosed by three pairs of transmitting and receiving ends, which can be considered as a larger drilling hole. The angular difference between θ and $\theta^{*}$ at the three clock positions is 51.85°, 57.44°, and 54.81°, respectively. The theoretical value of this angular difference is 60°. So the maximum deviation between the direction of the principal stress measured by the ultrasonic and blind hole methods is 8.15° (13.6%). This research demonstrates the success of the plane-stress measurement method, which relies on tri-directional ultrasonic waves, in determining the principal stress and its direction on pipe walls.

### 4.3. Influencing Factors of the Ultrasonic TOF

The increase of coupling pressure will reduce the thickness of the couplant, reduce the ultrasonic propagation path to a certain extent, and then affect the ultrasonic TOF measurement results, which is an important reason for the large error when using the hand pressure probe. Therefore, it is necessary to fix a stable coupling pressure at the time of measurement. In order to investigate the effect of the pressure applied directly above the probe on the ultrasonic TOF, a vertical pressure is applied to the ultrasonic probe and the ultrasonic signal response is measured using a tri-directional ultrasonic probe.

**Figure 7:**
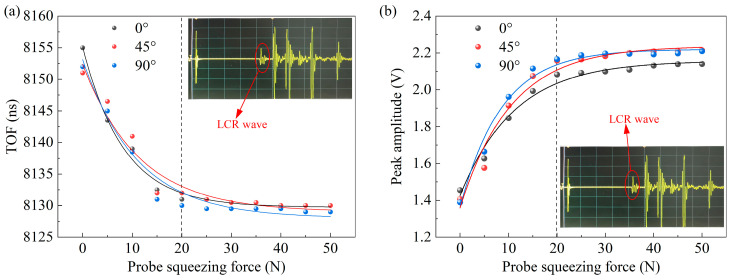
Relationship curve between ultrasonic LCR wave signals and probe extrusion pressure: (**a**) Results of the TOF measurement. (**b**) Results of peak amplitude of the LCR wave.

The coupling pressure and the corresponding ultrasonic signal response measurements are shown in Figure 7. It can be concluded that as the probe squeezing pressure increases, the ultrasonic TOF decreases, exhibiting a maximum reduction of 25 ns. In contrast, the LCR wave amplitude shows a positive correlation with pressure, with a maximum increase of 0.8 V. The probe squeezing pressure is in the range of 0–20 N, and both of them change drastically with the pressure. When the probe squeezing pressure reaches 20 N, the changes in TOF and wave amplitude begin to level off, and the relative changes of both are less than 10% in the range of 20–50 N. Therefore, in order to ensure the reliability of the ultrasonic TOF measurement results of this method, a constant pressure of at least 20 N on the probe specimen is required for the measurement of plane stress.

**Figure 8:**
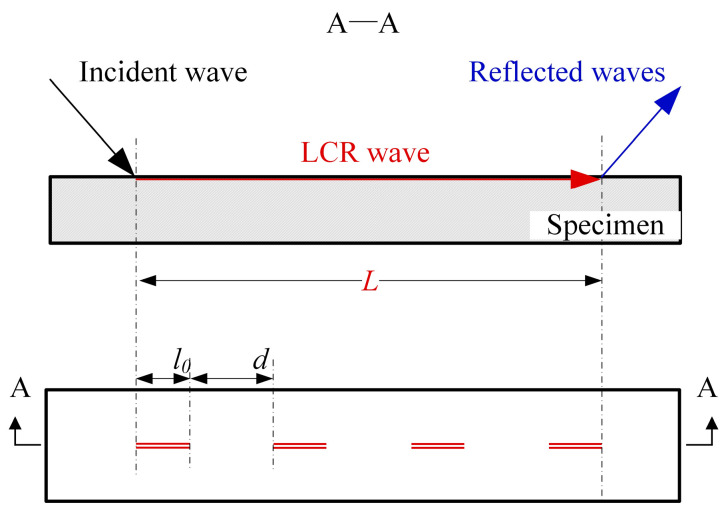
Measurement scheme of the surface roughness and ultrasonic TOF.

In order to study the influence of different surface roughness on the ultrasonic measurement results, different sandpapers are used to polish the X80 spiral-welded pipe calibration steel specimens, and the surface roughness and ultrasonic sound of the specimens are measured. As demonstrated in Figure 8, the surface roughness evaluation length l0 is 2.5 mm, and the transmitting and receiving distance *L* of the probe is 22 mm, so four roughness measurement points are set at equal intervals within the ultrasonic propagation distance, and the distance d between the two adjacent points is 4 mm. The average value of the four roughness measurements is taken as the effective value of the roughness corresponding to the ultrasonic measurement point. The specimen is polished successively with 100-, 150-, and 200-mesh sandpaper, with surface roughness and ultrasonic transit time measured 100 times after each polishing step. The surface roughness Ra is measured using a TIME3202 (Beijing TIME High Technology Ltd., Beijing, China) roughness meter. Table 4 shows the measurement results of the mesh and roughness of the sandpaper.

**Figure 9:**
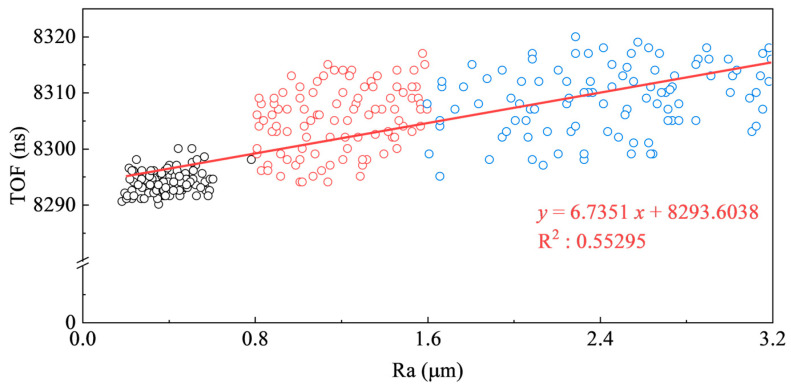
Relationship between TOF and surface roughness.

Effective values of roughness and the corresponding ultrasonic measurements are shown in Figure 9. The black circle indicates the ultrasonic TOF measurement results when the surface roughness Ra of the measurement point is less than 0.8; similarly, the red and blue circles represent the results for surface roughness values between 0.8 and 1.6, and 1.6 and 3.2, respectively. All measurements of Ra are taken along the direction of ultrasonic propagation. The results demonstrate that increased surface roughness leads to longer measured ultrasonic TOF. The ultrasonic TOF measurement results after sanding with fine sandpaper are relatively stable, and the deviation of the ultrasonic TOF measurement results is not more than 10 ns. The ultrasonic TOF measurement results after sanding with coarse sandpaper fluctuate more. This is because the rougher the surface, the greater the difference in profile height and spacing, resulting in a different degree of energy attenuation when ultrasonic waves are refracted and reflected at the interface, so the ultrasonic TOF measurements are more discrete. Therefore, in order to ensure the reliability of the ultrasonic TOF measurement results of this method, it is necessary to use 200 mesh or above sandpaper to polish the surface to be measured to ensure that the surface roughness Ra < 0.8.

## 5. Conclusions

In this paper, a nondestructive method for measuring the plane stress of anisotropic spiral-welded pipe is newly proposed, which utilizes a tri-directional LCR wave combined with ultrasonic TOF measurements. A tri-directional ultrasonic LCR wave probe is developed to capture the TOF along the 0°, 45°, and 90° directions relative to the pipeline axis under the same coupling condition. The principal stress level and orientation of X80 steel under uniaxial tensile stress are measured through the stress coefficient calibration examination. The ensuing conclusions are as follows:

➣ The principal stress obtained by the ultrasonic method is closer to the engineering stress than that obtained from the coercivity method.
➣ In varying stress conditions, the principal stress’s level and orientation variation are less than 20.5 MPa and 1.85°, in that order. The off-line pipe stress is highly consistent with that obtained by the hole-drilling method, which verifies that the improved ultrasonic method is more suitable for in-service pipelines.
➣ The stability of the TOF measurement requires that the probe pressure is not less than 20 N and the surface roughness is less than 0.8 μm.

This study provides a feasible technique for non-destructive measurement of plane stress in anisotropic spiral-welded pipelines, which has potential application prospects in the health monitoring of in-service pipelines.

## Figures and Tables

**Table 1:** Purpose of the experiment.

Specimen Processing Direction	Specimen Number	Purpose of the Experiment
0°	exp_1, exp_2	Calibration stress factor
	exp_3	Verification stress factor
60°	exp_4, exp_5	Calibration stress factor

**Table 2:** Combination of the calibration status and detection angle.

Calibration State	θ	ω	Stress Coefficient Expression
I	60°	60°	$T=\left(-p_{1}+p_{2}+p_{3}-\frac{1}{2}p_{4}\right)\sigma$
II	60°	30°	$T=\left(p_{2}+\frac{1}{2}p_{3}-p_{4}\right)\sigma$
III	0°	0°	$T=\left(2p_{1}+p_{2}+p_{3}+p_{4}\right)\sigma$
IV	0°	90°	$T=\left(p_{2}-p_{3}-p_{4}\right)\sigma$

**Table 3:** Stress coefficient calibration experimental data.

Calibration Experiments	Specimen Number	Δ*T*/*σ* [ns/MPa]
I	exp_1	0.05871
II	exp_2	−0.02737
III	exp_4	0.03678
IV	exp_5	−0.02868

**Table 4:** Roughness measurement of the specimen.

Grit of Sandpaper	Distribution of Ra
200	Ra < 0.8
150	0.8 ≤ Ra < 1.6
100	1.6 ≤ Ra < 3.2

## Data Availability

The original contributions presented in this study are included in the article. Further inquiries can be directed to the corresponding author.
